# Gluten-free diet may improve obstructive sleep apnea-related symptoms in children with celiac disease

**DOI:** 10.1186/s12887-018-1039-5

**Published:** 2018-02-07

**Authors:** Anat Yerushalmy-Feler, Riva Tauman, Ari Derowe, Eran Averbuch, Amir Ben-Tov, Yael Weintraub, Dror Weiner, Achiya Amir, Hadar Moran-Lev, Shlomi Cohen

**Affiliations:** 1 Pediatric Gastroenterology Unit, “Dana-Dwek” Children’s Hospital, Tel Aviv Sourasky Medical Center, Tel Aviv, Israel; 2 Pediatric Sleep Center, “Dana-Dwek” Children’s Hospital, Tel Aviv Sourasky Medical Center, Tel Aviv, Israel; 3 Pediatric ENT Unit, “Dana-Dwek” Children’s Hospital, Tel Aviv Sourasky Medical Center, Tel Aviv, Israel; 4 Sackler Faculty of Medicine, Tel Aviv University, Tel Aviv, Israel

**Keywords:** Celiac disease, Gluten-free diet, Obstructive sleep apnea, Pediatrics

## Abstract

**Background:**

Enlarged tonsils and adenoids are the major etiology of obstructive sleep apnea (OSA) in children. Lymphatic hyperplasia is common to both OSA and celiac disease. We aimed to investigate the effect of a gluten-free diet on OSA symptoms in children with celiac disease.

**Methods:**

Children with celiac disease aged 2–18 years were prospectively recruited before the initiation of a gluten-free diet. Children with negative celiac serology who underwent gastrointestinal endoscopies for other indications served as controls. All participants completed a validated OSA-related symptoms questionnaire and the pediatric sleep questionnaire (PSQ) at baseline and 6 months later.

**Results:**

Thirty-four children with celiac disease (mean age 6.6 ± 3.5 years) and 24 controls (mean age 7.3 ± 4.6 years, *P* = 0.5) were recruited. There were no significant differences in gender, body mass index or season at recruitment between the two groups. The rate of positive PSQ scores was higher (more OSA-related symptoms) in the control group compared to the celiac group, both at recruitment and at the 6-month follow-up (33.3% vs. 11.8%, *P* = 0.046, and 16.7% vs. 0, *P* = 0.014, respectively). PSQ scores improved significantly in both groups at the 6-month follow-up (*P* < 0.001 for both). Improvement was significantly higher in the celiac group compared to controls (0.1 ± 0.09 vs.0.06 ± 0.06, respectively, *P* = 0.04).

**Conclusions:**

Children with celiac disease had fewer OSA-related symptoms than controls, but the degree of improvement following the initiation of a gluten-free diet was significantly higher. These findings suggest that a gluten-free diet may improve OSA-related symptoms in children with celiac disease.

## Background

Celiac disease is a chronic immune-mediated systemic disorder caused by a permanent sensitivity to gluten and related proteins in genetically susceptible individuals. Its prevalence is approximately 1% of the general population [1, 2]. Mesenteric lymphadenopathy is a potential part of the clinical course of celiac disease, with resolution after the institution of a gluten-free diet [3, 4]. Celiac disease was also associated with mesenteric lymph node cavitation syndrome [5].

Obstructive sleep apnea (OSA) syndrome is characterized by recurrent episodes of upper airway obstruction during sleep, associated with intermittent hypoxia, hypercapnia and sleep fragmentation [6]. The prevalence of OSA in children is up to 3% in several epidemiological studies [7]. The incidence peaks in children from 2 to 8 years of age [8]. The major etiology for OSA in children is adenotonsillar hypertrophy [9, 10]. In addition, children with OSA may have hypertrophy of the lymphoid tissues in other regions of the airways, such as the deep cervical lymph nodes [11, 12]. Recent studies have indicated that a potential part of the pathophysiology of OSA is localized inflammation in the nasopharyngeal area, including an increase in inflammatory cell proliferation, particularly T-cell lymphocytes [13, 14].

Celiac disease and OSA share common features of lymphatic hyperplasia and local inflammation. A recent study by Parisi et al. [15] showed an increased prevalence of sleep-disordered breathing in celiac patients, with resolution of the symptoms with the introduction of a gluten-free diet. Considering the common feature of lymphatic hyperplasia/local inflammation in both disorders, we aimed to investigate the prevalence of OSA in children with celiac disease and to define the effect of a gluten-free diet on OSA symptoms in celiac disease.

## Methods

All study procedures were approved by the institutional review board of the Tel Aviv Medical Center (Helsinki Committee), and parental informed consent was obtained for all participants.

### Sample and procedure

The study group included children aged 2 to 18 years who were diagnosed as having celiac disease and were recruited between December 2014 and September 2015 at the Pediatric Gastroenterology Unit in “Dana-Dwek” Children’s Hospital of the Tel Aviv Sourasky Medical Center. All patients were prospectively recruited after diagnosis, before the initiation of a gluten-free diet. The diagnosis of celiac disease was based on the combination of celiac-related symptoms, a positive serology (anti-tissue transglutaminase levels [TTG] > 10.0 U/mL) and a characteristic histology according to the European Society for Pediatric Gastroenterology, Hepatology, and Nutrition (ESPGHAN) guidelines for the diagnosis of celiac disease [16]. Healthy children who underwent upper gastrointestinal endoscopy for other indications and who had a documented negative celiac serology during the past 3 months and a normal duodenal histology served as controls. The groups were matched for age, sex and season of referral. Children with congenital anomalies, developmental delay or other chronic medical conditions were excluded, as were children that had received medical/surgical treatment for OSA.

### Measures

We collected demographic data including age and gender, medical history, height, weight and body mass index (BMI), clinical symptoms, anti-TTG levels at baseline and 6 months after initiation of a gluten-free diet, histological findings and Marsh scores. The BMI Z score was calculated as well [17].

All parents completed the sleep-related breathing disorders scale of the pediatric sleep questionnaire (PSQ) at the time of recruitment (PSQ1) and 6 months later (PSQ2), i.e., prior to and 6 months after the initiation of a gluten-free diet for the celiac group [18]. The pediatric sleep questionnaire is a well validated symptom inventory that includes 22 items on snoring, apneas, daytime sleepiness, and inattentive/hyperactive behavior [18]. Responses are “yes” = 1, “no” = 0, or “don’t know” (considered missing). The mean response to non-missing items is the total score which can vary from 0 to 1. Higher scores indicate more sleep disordered breathing (SDB)-related symptoms [18]. A threshold of 0.33, indicating that 33% of symptom-related items are positive, is considered a positive screen for pediatric SDB [18]. Subscales within the PSQ include a 4-item sleepiness scale, a 4-item snoring scale, and a 6-item inattention and hyperactivity scale derived from the *Diagnostic and Statistical Manual of Mental Disorders, Fourth Edition Criteria for Attention-deficit/hyperactivity Disorder* [19]. Since its development, this scale has been used in a variety of research settings [18, 20]. For the purposes of the current study, we analyzed the total score, as well as the snoring, sleepiness and the inattentive/hyperactive behavior subscales.

### Data and statistical analysis

Analyses were performed with SPSS (version 21.0; SPSS Inc. Chicago, IL). Between-group comparisons of non-normally distributed continuous parameters (age, BMI and PSQ scores) were conducted with the non-parametric Wilcoxon test. Comparisons between pre- and post-intervention were conducted with paired-tests. All reported *P*-values were 2-tailed with statistical significance set at < 0.05.

## Results

### Participants

Thirty-four children with celiac disease (mean age 6.6 ± 3.5 years) and 24 controls (mean age 7.3 ± 4.6 years, *P* = 0.5) were included. Fifteen patients (44.1%) in the celiac group and 14 patients (58.3%, *P* = 0.29) in the control group were males. There was no significant difference in the BMI z scores between the two groups (Table 1).

**Table 1:** Demographic parameters of the celiac and control groups

	Celiac group *N* = 34	Control group *N* = 24	*P* value
Age, y	6.6 ± 3.5	7.3 ± 4.6	0.5
Male gender	15 (44.1%)	14 (58.3%)	0.29
Body mass index (z-score)	−0.4 ± 1.07	−0.9 ± 2.6	0.3
Recruitment in winter (%)	67.6%	58.3%	0.48
Time interval between questionnaires (months)	5.94 ± 1.25	5.95 ± 0.92	0.97

### Celiac symptoms

The main symptoms in the celiac group were abdominal pain and growth retardation (9 patients, 26.5% for both symptoms), anemia (5 patients, 14.7%) and diarrhea (4 patients, 11.7%). Celiac serology was performed as part of screening (first-degree relatives of celiac patients) in seven asymptomatic patients (20.5%). The mean anti-TTG antibodies level in the celiac group at diagnosis was 275 ± 34 U/ml (range 17–800 U/ml) and 23 ± 23 U/ml (range 0–76 U/ml) 6 months after the initiation of a gluten-free diet. Sixteen patients (47%) had normal macroscopic appearance of the duodenum, while 18 patients (53%) had macroscopic findings consistent with celiac disease, mainly duodenal scalloping. The duodenal histology of all patients except one demonstrated villous atrophy, crypt hyperplasia and an increased number of intraepithelial lymphocytes, consistent with Marsh 3 celiac disease. One patient had findings consistent with Marsh 2 celiac disease.

### Controls

The control group included 24 patients. Twelve of them (50%) had abdominal pain with a normal gastric histology, 4 (16.7%) had Helicobacter pylori-positive gastritis, 2 (8.3%) had Helicobacter pylori-negative gastritis, 3 (12.5%) presented with vomiting (2 with findings consistent with esophagitis and 1 with normal histology), and 3 (12.5%) presented with failure to thrive (FTT) with a normal histology. All patients in the control group had normal anti-TTG levels as well as a normal duodenal histology.

### PSQ

The average time interval between the two PSQ was 5.94 ± 1.25 months in the celiac group and 5.95 ± 0.92 months in the control group (*P* = 0.97). Most of the patients were recruited and filled out the first PSQ in the winter (November to March, 67.6 and 58.3% in the celiac and control groups, respectively; *P* = 0.48), and completed the second PSQ in a non-winter season (April to October, 79.4 and 75% in the celiac and control groups, respectively; *P* = 0.69).

The rate of positive PSQ scores was higher in the control group compared to the celiac group, both at study onset and at follow-up. Four out of 34 patients (11.8%) in the celiac group and 8 out of 24 patients (33.3%) in the control group (*P* = 0.046) had a positive PSQ score (≥0.33) at baseline. Six months later, no patient in the celiac group and 4 out of 24 (16.7%) patients in the control group had a positive PSQ score (*P* = 0.014).

The total PSQ scores were significantly lower in the celiac group compared to controls at both baseline and at the 6-month follow-up. The PSQ subscale scores (snoring and inattention and hyperactivity scales) were also significantly higher for the controls compared to the celiac group both at baseline and at follow-up (Table 2).

**Table 2:** The pediatric sleep questionnaire (PSQ) scores in the celiac and control groups

	Celiac group			Control group			*P* value	
Subscale	PSQ1	PSQ2	*P* value	PSQ1	PSQ2	*P* value	PSQ1 (celiac vs controls)	PSQ2 (celiac vs controls)
Snoring	0.07 ± 0.13	0.010.04±	0.01	0.18 ± 0.22	0.16 ± 0.2	0.180	0.022	< 0.001
Sleepiness	0.2 ± 0.23	0.09 ± 0.15	0.001	0.2 ± 0.27	0.11 ± 0.19	0.023	0.75	0.81
Attention/behavioral	0.19 ± 0.29	0.08 ± 0.17	0.001	0.34 ± 0.28	0.29 ± 0.25	0.035	0.014	< 0.001
Total PSQ	0.17 ± 0.14	0.07 ± 0.06	< 0.001	0.26 ± 0.15	0.2 ± 0.11	< 0.001	0.022	< 0.001

**Fig. 1:**
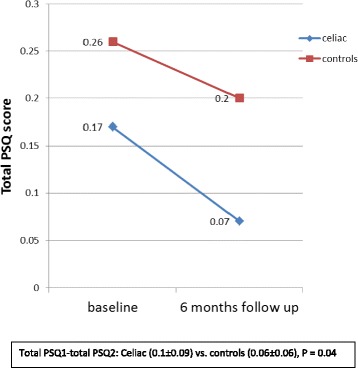
Average total pediatric sleep questionnaire (PSQ) scores at baseline and at the 6-month follow-up

As displayed in Fig. 1, the total PSQ scores improved significantly in both groups at the 6-month follow-up compared to baseline (*P* < 0.001 for both groups). There was improvement in all three subscales of the PSQ (snoring, sleepiness and attention/behavioral subscales) in the celiac group and in two subscales (sleepiness and attention/behavioral subscales) in the control group. The degree of improvement (total PSQ1–total PSQ2) was significantly higher in the celiac group compared to the controls (0.1 ± 0.09 vs. 0.06 ± 0.06, respectively, *P* = 0.04, Fig. 1). As expected, there was a significant correlation between the total PSQ1 and the total PSQ2 (*r* = 0.87, *P* < 0.0001). No correlation was found between total PSQ scores (at baseline or at follow-up) and age, BMI Z score and anti-TTG levels.

Linear regression analysis with PSQ2 as a dependent variable and group assignment (celiac vs. controls), age, BMI z score and season as independent variables revealed that only group assignment was a significant predictor for PSQ2 score *(P < 0.001).* Since FTT is also a manifestation of OSA, we re-analyzed the data after excluding the three patients with FTT and found no differences in the results.

## Discussion

This is the first prospective controlled trial that investigated the prevalence of OSA symptoms in children with celiac disease and the effect of a gluten-free diet on these symptoms. In this cohort of children, the rate of abnormal PSQ scores, which is a symptom inventory for childhood OSA, was significantly lower among children with celiac disease compared to age- and gender-matched controls. The PSQ scores were lower in the celiac group compared to the controls, while improvements in PSQ scores were found in both groups 6 months later, and they were even greater in the celiac group. Our findings are in contrast to those of Parisi et al. [15] who demonstrated an increased prevalence of sleep-disordered breathing in celiac patients. Their study, however, used a different tool for the assessment of sleep and their sample size (*n* = 19) was smaller compared to that of the present study.

The PSQ total scores and the rate of abnormal PSQ scores were significantly lower in the celiac group compared to controls both at baseline and at follow-up. Both the snoring subscale and the attention/behavioral subscale scores were lower in the celiac group compared to controls. These differences cannot be explained by age, BMI or season of assessment since there were no differences in these parameters between the two groups. One possible explanation for these findings is an increased prevalence of OSA symptoms in the control group due to factors that were not measured in the current study, such as prematurity, atopic diseases, parental smoking or family history of OSA. We speculate, however, that such factors would potentially impact both groups.

The improvement found in PSQ scores in both groups at the 6-month follow-up may be explained by the natural history of OSA, which tends to improve with age. Indeed, the age range of our cohort is toward the upper limit of the peak incidence of OSA in children. It is possible that some children experienced improvement or even resolution of OSA symptoms during this 6-month period. Another explanation is the change in season. Most of our participants were recruited in the winter and so their follow-up assessment was in the spring/summer. As shown in earlier reports, OSA symptoms may vary with season, and winter is usually associated with a worsening of OSA symptoms [21–23].

Although PSQ scores were lower in the celiac group in our study, the degree of improvement in OSA-related symptoms was significantly higher for those children compared to controls. This finding suggests that a gluten-free diet may play a role in this improvement since there was no difference in the time interval or seasonality between the two groups. This finding is in agreement with that of Parisi et al. [15] who also showed resolution of sleep-disordered breathing with a gluten-free diet. A potential explanation for this finding is improvement in the lymphatic hyperplasia that is associated with celiac disease and that contributes to OSA in these patients. The significant improvement in OSA in celiac patients after the institution of a gluten-free diet raises the question of whether a gluten-free diet might have the potential to improve OSA symptoms in children with celiac and thus serve as an adjuvant therapy for OSA or even replace adenotonsillectomy as the first line of treatment for OSA for those children.

Our study is based on a well-established tool for identifying OSA symptoms in the pediatric population. It is the first prospective controlled trial that demonstrated a possible benefit from a gluten-free diet on OSA in children with celiac disease. However, the results of the present study should be interpreted in the context of several limitations. First, our data are based on a questionnaire and not on objective measures of OSA, such as polysomnography. Second, we did not assess adenoid and tonsil size. In addition, the control group is relatively small due to exclusion of patients with chronic medical conditions, but the results were obtained in this cohort nevertheless reached a level of significance. Moreover, three patients in the control group had FTT, which itself is a potential manifestation of OSA. We decided to include these three patients in the study since they had similar PSQ scores as those of the control group. Moreover, as mentioned earlier, the analysis of the data after excluding these three patients did not change the results of the study.

In summary, children with celiac disease were found to have fewer OSA-related symptoms, but the degree of improvement following the initiation of a gluten-free diet was significantly higher in the celiac group compared to the controls. Our findings suggest that a gluten-free diet may improve OSA-related symptoms in children with celiac disease. Further studies are needed to determine whether a gluten-free diet has the potential to reduce the need for adenoidectomy and/or tonsillectomy in children with celiac and OSA.

## Conclusions

Children with celiac disease had fewer OSA-related symptoms than controls, but the degree of improvement following the initiation of a gluten-free diet was significantly higher. These findings suggest that a gluten-free diet may improve OSA-related symptoms in children with celiac disease.

## Abbreviations

BMI: Body mass index
ESPGHAN: European Society for Pediatric Gastroenterology, Hepatology, and Nutrition
FTT: Failure to thrive
OSA: Obstructive sleep apnea
PSQ: Pediatric sleep questionnaire
SDB: Sleep disordered breathing
TTG: Tissue transglutaminase
